# Standardization and Validation of Scalp Surface Area Measurement and Assessment of Interchangeability Between Two Independent Methodologies for Head Crown Area and Total Hair Count Measurement

**DOI:** 10.7759/cureus.104173

**Published:** 2026-02-24

**Authors:** Maheshvari N Patel, Nayan Patel

**Affiliations:** 1 Clinical Research, NovoBliss Research Private Limited, Ahmedabad, IND; 2 Pharmacology, Swaminarayan University, Ahmedabad, IND; 3 Clinical Research Operations, NovoBliss Research Private Limited, Ahmedabad, IND

**Keywords:** alopecia, hair count, hair loss, image-pro software, method validation, phototrichogram, scalp surface area, standardization

## Abstract

Background

Hair loss significantly affects physical appearance and psychological well-being, leading to increased use of hair growth and hair loss prevention treatments. Accurate and reproducible assessment of scalp and head crown surface area and hair count is essential for evaluation in clinical and cosmetic clinical studies with hair care products.

Objective and method

This prospective, single-visit standardization and validation study included adult participants aged 18 to 55 years. Scalp surface area measurements were performed in 25 adult participants using a calibrated measuring tape. Head crown (vertex) surface area measurements, including the frontal and mid-scalp regions, were performed in 25 adult participants using a calibrated measuring tape and photographic image analysis with Image-Pro software. For hair count assessment, global 90° head photographs obtained from in-house data of 50 participants were analyzed using phototrichogram (PTG)-based CASLite NOVA and Image-Pro software. Agreement between the measurement methods was evaluated using appropriate method-comparison statistical analyses.

Result

The scalp surface area mean was 492.36 ± 30.97 cm².* *For head crown, including frontal and mid-scalp area measurements, Bland-Altman analysis demonstrated minimal systematic bias with acceptable limits of agreement (LoA) between the calibrated measuring tape and Image-Pro analysis. The intraclass correlation coefficient (ICC) of 0.75 indicates good reliability and consistency between the two measurement methods, while the concordance correlation coefficient (CCC) of 0.74 reflects moderate agreement when both precision and accuracy are considered. Hair count assessment using CASLite NOVA and Image-Pro analysis also showed good agreement in hair counts, with small average differences and expected LoA.

Conclusion

This standardization and validation study demonstrated acceptable agreement between measure-tape-based calculated and image-based methods for head crown area assessment, including the frontal and mid-scalp regions. Additionally, acceptable agreement was observed between CASLite NOVA and Image-Pro software for hair count evaluation. These findings support the interchangeable use of measure-tape-based and image-based methods for head crown area measurement, as well as the reliability of CASLite NOVA and Image-Pro-based analyses for hair count assessment in hair care product testing and clinical and cosmetic hair research.

## Introduction

Hair loss has a multifactorial impact, affecting not only an individual’s physical appearance but also psychological well-being. Several studies have demonstrated that hair loss may lead to reduced self-esteem, social anxiety, and diminished quality of life, thereby increasing the demand for effective hair loss prevention strategies and promotion of new hair. Consequently, a wide range of therapeutic and cosmetic products has emerged in the market, ranging from pharmaceutical interventions to cosmetic hair care formulations aimed at improving hair parameters [1].

With the growing interest in hair loss management, standardized and reproducible evaluation methods of hair parameters have become increasingly important. Contemporary hair research employs various methods for assessing hair and scalp characteristics, broadly classified into non-invasive, semi-invasive, and invasive techniques. Among these, non-invasive methods are preferred for clinical and cosmetic studies due to their safety, repeatability, and suitability for assessments. Global photography and PTG-based techniques are considered among the most valuable and widely accepted tools for clinical hair evaluation, as they allow standardized documentation and quantitative analysis without causing discomfort to participants [2-4].

PTG techniques enable imaging of a defined region, facilitating quantitative assessment of hair count. These methods are particularly suitable for clinical studies due to their ability to provide reliable and reproducible measurements [5,6]. In addition to hair count, accurate measurement of scalp surface area and clinically relevant regions, such as the head crown (vertex), including the frontal and mid-scalp, is critical for standardized hair parameter analysis.

Despite the availability of multiple assessment tools, variability may arise due to differences in measurement techniques, operator handling, and analytical algorithms. Importantly, a high correlation between two measurement methods does not necessarily imply that they can be used interchangeably. Therefore, agreement-based validation, rather than correlation alone, is required to determine whether different methodologies yield comparable results suitable for standardized use. Statistical approaches such as Bland-Altman analysis are used for evaluating agreement and identifying systematic bias between methods.

In the absence of standardized and validated measurements, variability in head crown, including frontal and mid-scalp region surface area and hair count assessment, may compromise data reliability, limit comparability across studies, and affect the interpretation of treatment outcomes. Standardization and validation of methodologies are therefore essential to ensure consistency, accuracy, and reproducibility in research. Establishing agreement between methodologies enables confidence in their interchangeable application. From a pharmacological and cosmetic product development perspective, reliable quantification of scalp surface area, head crown area, and hair count is fundamental for accurate dose exposure assessment, efficacy evaluation, and comparison of treatment outcomes across studies. In clinical investigations of hair growth-promoting or hair loss-preventive interventions, outcome measures such as hair density, total hair count, and region-specific scalp coverage directly influence the interpretation of product performance and therapeutic benefit. Inconsistent or non-standardized measurement approaches may lead to variability in efficacy and limit translational relevance from clinical evaluation to real-world use. There is a need for validated, reproducible, and well-defined assessment methodologies to support efficacy and ensure comparability across products. Establishing standardized and validated measurement techniques is particularly important in cosmetic and topical pharmacology research, where small variations in the application area or hair count outcomes may substantially influence conclusions regarding product effectiveness.

The present study evaluates: I) to measure total scalp surface area using a calibrated measuring tape-based method. II) To evaluate agreement between calibrated measuring tape-based calculations and photographic image analysis (Image-Pro software) for head crown surface area, including frontal and mid-scalp region estimation. III) To assess agreement between PTG-based hair count measurement using CASLite NOVA and image-based hair count assessment using Image-Pro software.

## Materials and methods

Ethical conduct of the study

This study was conducted according to the International Council for Harmonisation-Good Clinical Practice (ICH-GCP) and the Declaration of Helsinki to ensure the protection of the subjects' rights, safety, and well-being. Ethical approval for the study validation plan NB-VS002.01 was obtained from the ACEAS independent ethics committee on 03 Jan 2026, prior to the commencement of the study procedures (Approval Number: NB250062-NB-V). All participants provided written consent before enrollment in the study. The consent process included a detailed explanation of the study and the voluntary nature of participation.

Study design

This was a prospective, single-visit standardization and validation study conducted to evaluate scalp surface area measurement and agreement between different methodologies for head crown (vertex, including frontal and mid-scalp) surface area and hair count assessment. The study focused on comparing measure-tape-based calculated and photographic image-based techniques for head crown, including frontal and mid-scalp area measurement, and PTG-based CASLite NOVA with Image-Pro image analysis for hair count assessment, to determine their suitability for interchangeable use in clinical and cosmetic hair research. The study was conducted at NovoBliss Research Private Limited, Ahmedabad, India, a Central Drugs Standard Control Organization (CDSCO)-registered Contract Research Organization (CRO), where all study-related procedures were performed in accordance with ethical requirements.

Eligible participants included adults aged ≥18 years at the time of informed consent with visible scalp hair suitable for imaging and hair count analysis. Both male subjects and healthy non-pregnant, non-lactating female subjects were eligible for participation. All participants were required to be generally in good health, willing and able to comply with study procedures, and to provide written informed consent prior to participation. Participants were excluded if they had any active scalp disease or condition, a history of scalp surgery, scarring, or trauma that could interfere with accurate measurements, or the presence of implanted hair systems, wigs, or hair extensions.

To minimize variability due to hair volume or styling: I) Participants were instructed to attend the visit with clean, dry hair. II) Use of volumizing products, hairspray, mousse, gels, fibers, or styling agents was prohibited on the day of assessment. III) For participants with longer hair, hair was loosely secured posteriorly without tension to ensure full exposure of the crown region. IV) No hairstyles were permitted.

Method of standardization and methodology

Scalp Surface Area

**Figure 1:**
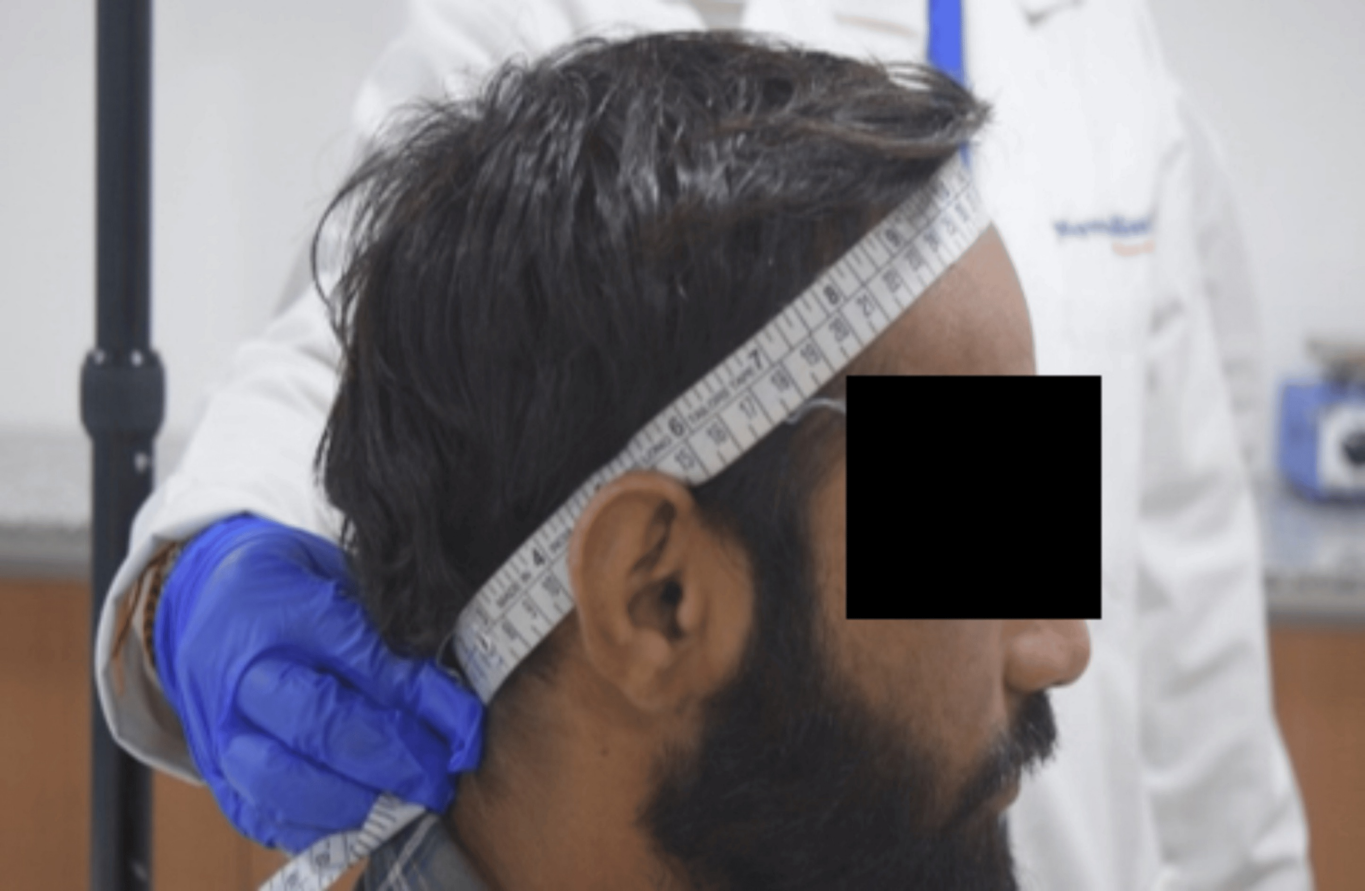
Scalp surface area measurement This figure demonstrated scalp surface area measurement by using calibrated measuring tape.

Scalp surface area measurements were performed using a calibrated measuring tape (Instrument ID NB/Measure Tap/038). Anatomical areas were predefined to ensure reproducibility. The anterior boundary was defined at the frontal hairline, the posterior boundary at the occipital hairline, and the lateral boundaries above the superior insertion of the auricles (temporal-parietal region) as shown in Figure 1. The scalp surface area was then estimated using a geometric method. The same investigator performed all measurements to minimize inter-operator variability. This assessment was conducted as a standalone objective to standardize total scalp surface area estimation using a reproducible manual approach (Figure 1).

Head Crown, Including Frontal and Mid-Scalp Area

**Figure 2:**
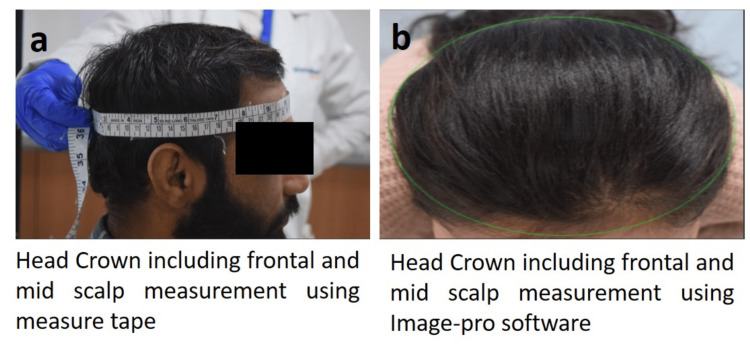
Head crown area measurement Figure 2 (a) demonstrates measurement of the head crown area using calibrated measuring tape, and (b) measurement of the head crown area using Image-Pro software.

The area of the head crown, including the frontal and mid-scalp, was assessed using two independent methodologies: calculation-based measurement with a calibrated measuring tape and photographic image analysis using a Nikon D3300 digital camera, and analyzed by Image-Pro software (Image-Pro, 10.0.13). The head crown region was wrapped with the tape snugly around the widest possible circumference, from the most prominent part of the forehead (1-2 fingers above the eyebrow) around to the widest part of the back of the head, as shown in Figure 2. Circumference was measured using a calibrated tape, and then a geometric formula was used to measure surface area. All photographs were obtained in a seated position using a chin-support table to maintain consistent head positioning and minimize positional variability during image capture. All images were captured using a fixed camera distance and standardized lighting conditions. Measurements were performed by the same trained investigator to minimize inter-operator variability (Figure 2).

Hair Count

**Figure 3:**
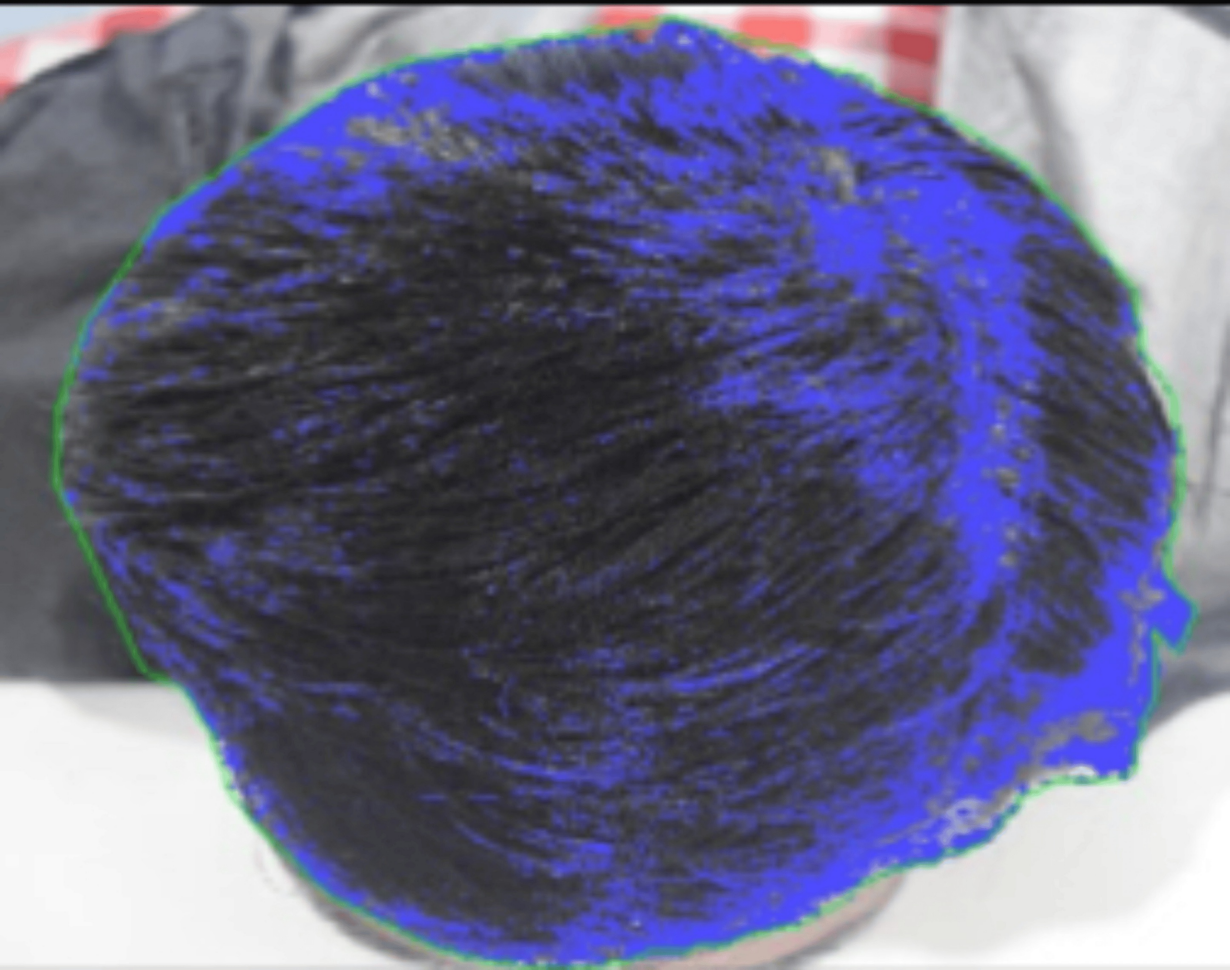
Hair count measurement Figure 3 demonstrated assessment of hair count from the head crown area, including frontal and mid-scalp regions, using Image-Pro software.

For hair count analysis, global photographs of the head crown and corresponding PTG were obtained from in-house data and analyzed using two methods: PTG-based analysis with CASLite NOVA and photographic image analysis using Image-Pro software. For hair count assessment, all photographs were captured at a standardized 90° angle to ensure uniformity and accuracy in image-based analysis. Hair count using CASLite NOVA was obtained within a predefined area, and then the total hair count for the defined area was calculated. Similarly, for Image-Pro analysis, a region of interest was selected based on defined anatomical boundaries, and total hair count was subsequently calculated for the defined area (Figure 3).

Statistical analysis

Descriptive statistics were used to summarize scalp surface area and hair count measurements obtained by each method. Continuous variables were expressed as number of observations (N), mean, standard deviation (SD), median, minimum, and maximum values.

Agreement between measurement methods was assessed using Bland-Altman analysis. For each paired measurement, the difference between the two methods was plotted against the mean of the measurements. The mean difference (bias) was calculated to assess systematic overestimation or underestimation between methods, and the 95% LoA was determined as the mean difference ±1.96 times the SD of the differences. Agreement was considered acceptable when the majority of observations fell within the LoA, and no clinically meaningful systematic bias was observed. Measurement variability and precision were further evaluated using the coefficient of variation (%CV) for each method. Lower %CV values were interpreted as indicative of greater measurement consistency. The ICC was calculated to assess the reliability between two methods. The CCC evaluates the overall agreement between two methods by accounting for both precision and accuracy.

All statistical analyses were performed using R software (version 4.2.2 or higher). Graphical representations, including Bland-Altman plots, were generated to support visual interpretation of agreement between methodologies.

Sample size calculation

A total of 25 participants were enrolled using the convenience sampling method; participants were selected based on accessibility and feasibility. This approach was suitable for the study's objective, which was to standardize and validate the assessment of agreement between different methods for measurement of the head crown area (including frontal and mid-scalp regions) and hair count, and no therapeutic interventions were used in this study. The selected sample size was sufficient for methodological validation, ensuring the reliability and consistency of the assessment techniques. All 25 participants successfully completed the study and were included in the final data analysis.

For hair count validation, 50 global head crown images with corresponding PTG obtained from in-house data were analyzed. This sample size was sufficient to evaluate the agreement between the two methods.

Study disposition

Study validation plan NB-VS002.01 was approved on 03 January 2026 by the ACEAS Independent Ethics Committee prior to study initiation. The study commenced on January 12, 2026, and January 13, 2026, and all 25 participants were enrolled, completed, and included in the final statistical analysis. None of the participants withdrew from the study. All study measurements were completed in accordance with the approved study validation plan, with no deviations reported.

## Results

Scalp surface area

Scalp surface area measurements were recorded for 25 participants. The measured values ranged from 447.29 to 592.52 cm². The mean scalp surface area across all participants was 492.36 ± 30.97 cm². These values are consistent with reported adult human scalp surface areas, which typically range from approximately 500 to 700 cm² [7,8].

Head crown area measurement

**Figure 4:**
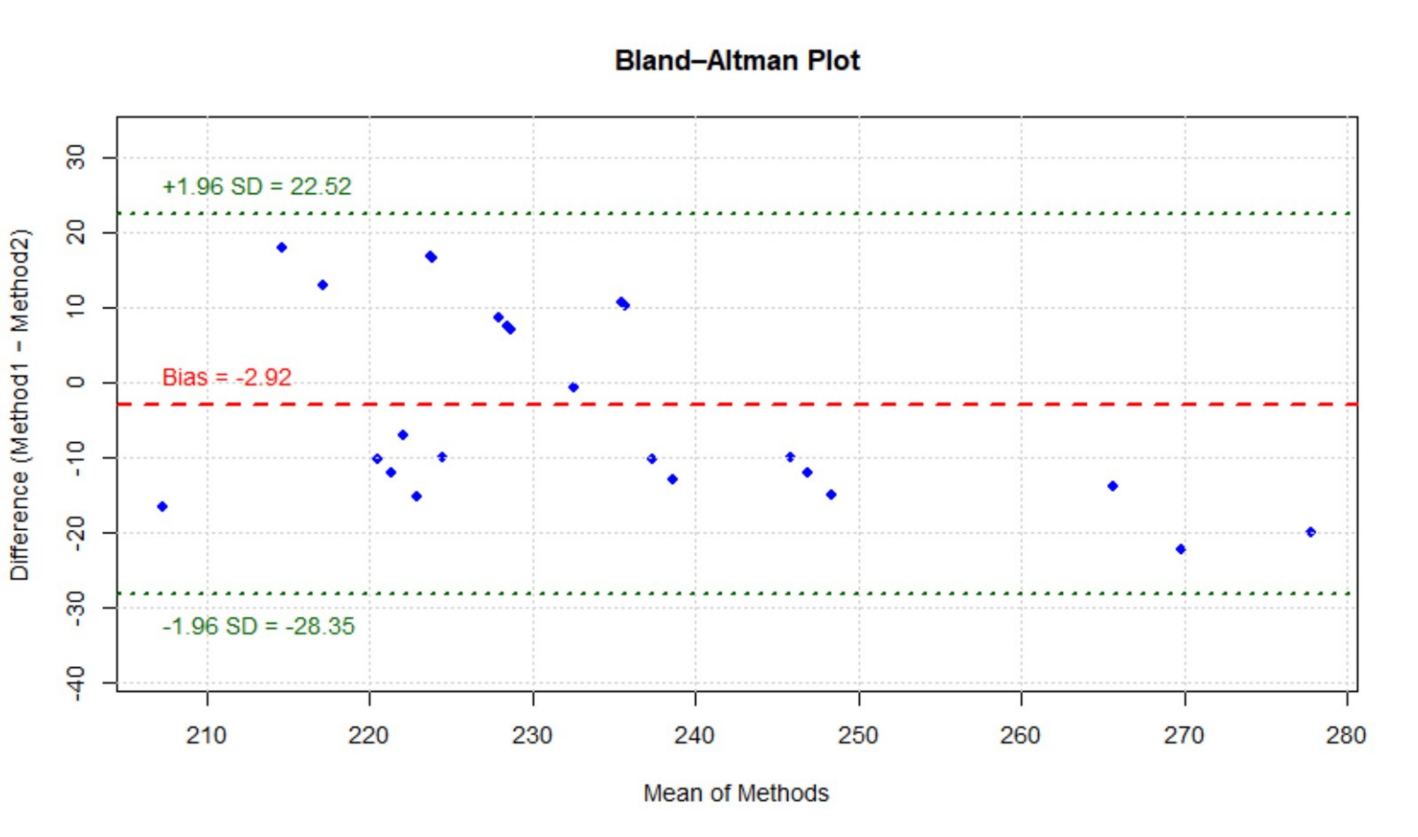
Bland–Altman plot for area measurement Figure 4 demonstrates the agreement between methods for measuring head crown area, including the frontal and mid-scalp regions, using calibrated measuring tape–based calculations and photographic image analysis performed with Image-Pro software.

Method agreement for the head crown area, including frontal and mid-scalp regions, was evaluated between calibrated measuring tape-based calculation and photographic image analysis using Image-Pro software. Bland-Altman analysis demonstrated a mean bias of -2.92, indicating a slight systematic underestimation of head crown, including frontal and mid-scalp area measurements obtained using the calibrated measuring tape, compared with Image-Pro analysis. The 95% LoA ranged from -28.35 to 22.52, with the majority of observations falling within these limits, indicating acceptable agreement between the two measurement methods (Figure 4).

The ICC between the two methods was 0.7527, indicating good reliability. This result reflects a high degree of consistency and reproducibility between calibrated tape and Image-Pro measurements across participants, supporting the findings of the Bland-Altman analysis.

The CCC, which evaluates both precision and accuracy relative to the line of perfect agreement, was 0.74, indicating moderate agreement between the two methods. This suggests that although some variability exists at the individual level, the overall concordance between the methods is acceptable.

Hair count measurement

Hair counts obtained using CASLite NOVA and Image-Pro analysis [9] were compared to evaluate agreement between the two methodologies. Bland-Altman analysis demonstrated good agreement between CASLite NOVA and Image-Pro analysis for hair count measurements. The mean difference (bias) between the two methods was -2,430.06 hairs, indicating that one method measured, on average, approximately 2,430 hairs fewer than the other. This difference was considered small relative to the overall magnitude of hair counts.

**Figure 5:**
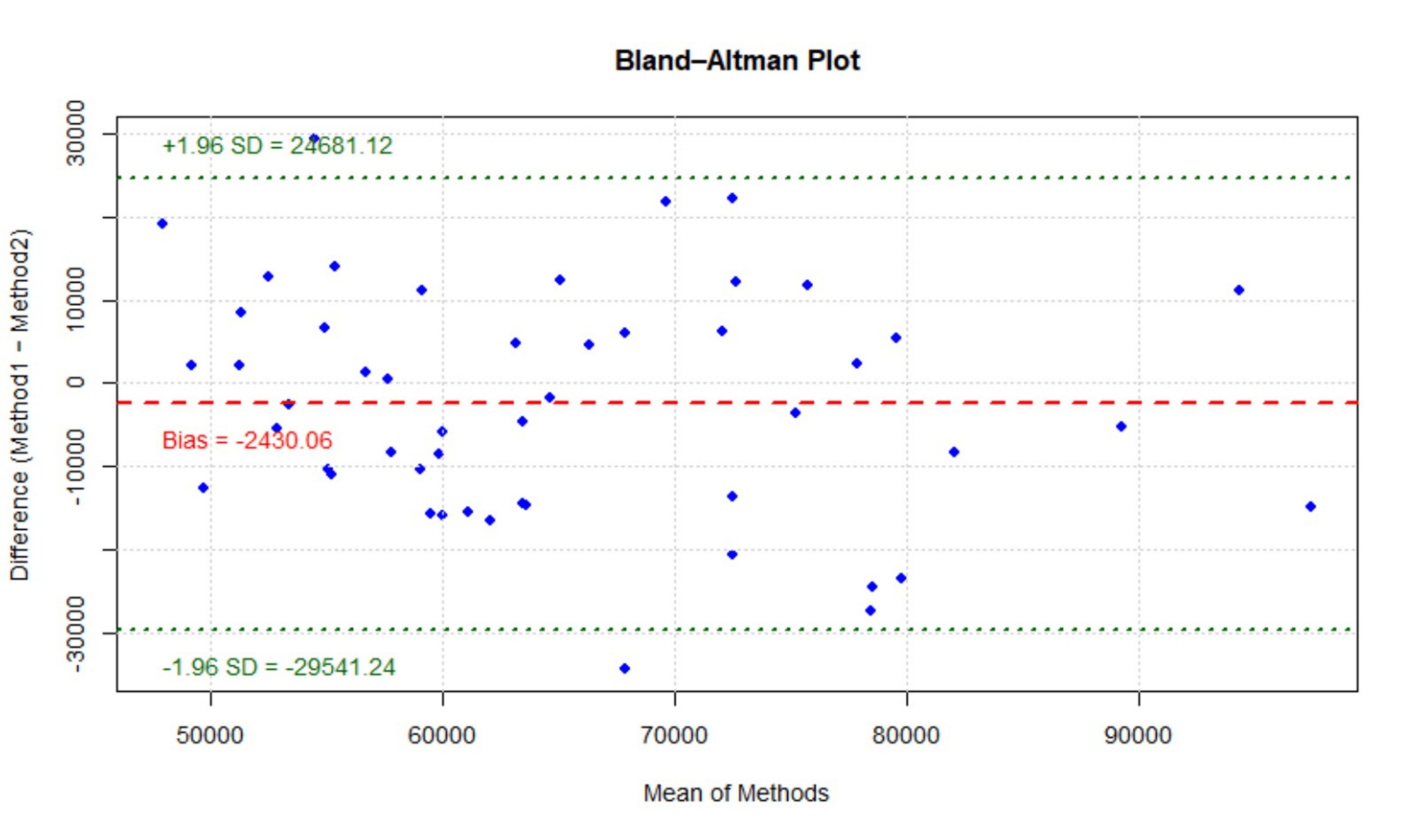
Bland-Altman plot for hair count assessment Figure 5 demonstrates the agreement between methods for hair count using CASLite NOVA and Image-Pro Analysis.

The 95% LoA ranged from -29,541.24 to 24,681.12 hairs, within which the majority of observations were distributed. Of the 50 measurements, 48 observations fell within the LoA, with only two values marginally outside the limits. No clear trend of proportional bias was observed across the range of hair counts (Figure 5).

Measurement precision was further evaluated using the %CV for each method. The mean hair count was 63,996 hairs for Image-Pro Analysis and 66,426 hairs for CASLite NOVA. The corresponding standard deviations were 12,534.76 and 14,643.13, respectively. The calculated %CV was 19.59% for Image-Pro Analysis and 22.04% for CASLite NOVA.

Both methods demonstrated acceptable levels of variability, with Image-Pro Analysis showing slightly lower relative variation compared to CASLite NOVA. However, the difference in %CV between the two methods was small and not considered sufficient to impact their practical interchangeability.

## Discussion

Hair loss has a well-documented impact on both physical appearance and psychological well-being, leading to increased interest in objective, reliable methods for evaluating hair and scalp parameters in clinical and cosmetic research [10]. The growing demand for hair loss prevention and hair growth-promoting interventions necessitates robust measurement methodologies that ensure consistency, reproducibility, and comparability across studies and assessment tools. According to published literature, it is mentioned that any single method is neither ideal nor feasible [2]. There are non-invasive, semi-invasive, and invasive techniques. Non-invasive techniques such as global photography and PTG-based assessments have been widely recognized as the most valuable tools for hair evaluation in clinical studies due to their repeatability and subject acceptability [2].

Standardization of measurement methodologies is particularly critical in clinical and cosmetic hair research, where treatment efficacy is often assessed using subtle changes in hair count or area measurement. Variability arising from inconsistent assessment techniques may interfere with outcome interpretation and reduce the reliability of efficacy results. Establishing validated methodologies that demonstrate agreement across different measurement techniques supports the harmonization of data generation and facilitates the broader applicability of research findings. Agreement-based validation plays a major role in determining whether different assessment techniques can be applied interchangeably.

In the present study, agreement between different methodologies for head crown, including frontal and mid-scalp region measurement and hair count assessment, was evaluated using appropriate method-comparison statistics. For head crown, including frontal and mid-scalp region measurements, comparison between calibrated measuring tape and photographic image analysis using Image-Pro software demonstrated acceptable agreement. Bland-Altman analysis revealed a small negative bias, and most observations lay within the 95% LoA. Reliability analyses further supported these results, with the ICC demonstrating good reliability and the CCC indicating moderate agreement, confirming consistency between methods. Similarly, hair count assessment using PTG-based CASLite NOVA and Image-Pro analysis showed acceptable agreement. PTG techniques are well-suited for clinical studies due to their reproducibility and non-invasive nature. Bland-Altman analysis demonstrated minimal systematic bias and acceptable LoA between the two hair count methods. Although small differences in absolute values were observed, these were minor relative to the overall measurement range and are unlikely to affect clinical interpretation or study outcomes.

The practical implications of this validation study are significant. Demonstrating agreement between measure-tape-based calculated and image-based area measurements allows flexibility in method selection. Similarly, validation of interchangeable hair count methodologies supports consistent outcome assessment. Nevertheless, to minimize within-subject variability, the use of the same assessment method for each subject across all study visits is recommended. This study has certain limitations. The study was conducted at a single research center, and another limitation of this study is that scalp surface area and head crown area measurements were derived using calibrated measuring tape-based measurements with calculated area estimation, which may not fully capture the complex three-dimensional curvature of the scalp. Incorporation of three-dimensional (3D) image-based analysis could provide a more precise and comprehensive assessment of scalp surface area by accurately mapping surface contours and minute anatomical variations that may not be adequately captured through manual or two-dimensional approaches. The use of 3D imaging techniques may also allow improved evaluation of regional variability and inter-individual differences in scalp morphology, thereby reducing potential measurement variability. Future studies integrating 3D imaging as an alternative or complementary methodology may further enhance measurement accuracy, standardization, and reproducibility, and contribute to more robust evaluation of scalp-surface area in clinical and cosmetic hair research.

## Conclusions

With the growing emphasis on objective assessment in hair loss management, the need for standardized and reproducible evaluation methodologies has become increasingly important. This standardization and validation study demonstrates that calibrated measuring tape-based assessment and photographic image analysis using Image-Pro software show acceptable agreement for head crown area measurement, including the frontal and mid-scalp regions, with minimal systematic bias and good reliability. Similarly, hair count assessment performed using PTG-based CASLite NOVA and Image-Pro analysis demonstrated comparable performance, with acceptable agreement and measurement consistency. Agreement analyses using Bland-Altman methodology for hair count assessment and Bland-Altman analysis in conjunction with ICC and CCC for area measurement confirm the comparability of the evaluated techniques. Collectively, these findings support the interchangeable use of the assessed methodologies for head area measurement, while indicating acceptable methodological agreement for hair count evaluation in clinical and cosmetic hair research.
